# Principles of Human Physiology, with Their Chief Applications to Pathology, Hygiene, and Forensic Medicine

**Published:** 1850-05

**Authors:** 


### ARTICLE II.

*Principles of Human Physiology, with their chief Aapplications to Pathology, Hygiene, and Forensic Medicine.*— By WILLIAM R. CARPENTER, M.D., F. R. S., F. G. S., Examiner in Physiology in the University of London; corresponding member of the American Philosophical Society, and of the National Institute of the United States; Lecturer on Physiology at the London Hospital Medical School. Fourth American Edition, with extensive additions and improvements by the Author. Two Plates and three hundred and four wood-cuts. pp. 750, 8vo. 1850.—Lea & Blanchard, Philadelphia. (From the Publishers.)

Altogether this is the best text book within the reach of the Medical student. Its author is a highly distinguished Anatomist and Physiologist, and his work gives evidence of extensive, patient, and discriminating research. The volume before us is a complete summary of all the modern discoveries and doctrines of the most distinguished physiologists, and is therefore superior, as a text book, or work of reference to any other now in the market.

The Author has, in our opinion, improved his work by the substitution of more rational views of the Nervous System, than those contained in the former edition. For however ingenious and plausible the theory of Marshal Hall, it lacks the exactness necessary to produce conviction in an inquiring and candid mind. The chapter on Generation has also undergone thorough revision and alteration, and may be regarded as containing the received doctrine on this interesting topic.

At the close of the volume is an appendix containing two short chapters; the one devoted to Phrenology, the other to Mesmerism. We are pleased to see on record in a standard medical work, accessible to the entire profession, a summary of the anatomical and physiological facts, which in our opinion, entirely invalidate the conclusions of the Phrenologists. The chapter on Artificial Somnambulism and Mesmerism, though not as full as we could desire, is yet very interesting, and suggestive to the physiologist and mental philosopher, of many important reflections.

There is one circumstance connected with the appearance of this volume, which is worthy of remark. This edition was specially prepared for the American market, the demand here having anticipated the exhaustion of the last English edition. We would infer from this, that medical books meet with a much more ready sale here, than in Great Britain. This being the case we regret that the demand cannot be supplied by native productions. The enterprising gentlemen who have brought out the present volume (and in praise of whom as medical publishers, too much cannot be said) would doubtless just as readily pay for an American as an English work; and although Dr. Carpenter's volume is a valuable one, we doubt not there are amongst us those who are capable of producing one equal to it in every respect. We hope therefore, that by the time the present edition shall have been exhausted, an American Author will have a volume prepared to replace it. M.

